# Whiteness and greenness metric tools for assessment of the spectrophotometric methods sustainability: mathematical manipulation of absorption spectra to resolve the interfering donepezil HCl and pentoxifylline in ethoniosomes

**DOI:** 10.1186/s13065-026-01759-4

**Published:** 2026-03-14

**Authors:** Mennah M. Abd El wahab, Samar Saad, Nahed El-Enany, Zeinab A. Sheribah

**Affiliations:** 1 Department of Pharmaceutical Analytical Chemistry, Faculty of Pharmacy, Sinai University (Arish branch), Arish, 45511 Egypt; 2 Department of Pharmaceutical Analytical Chemistry, Faculty of Pharmacy, Mansoura University, Mansoura, 35516 Egypt; 3 Department of Pharmaceutical Analytical Chemistry, Faculty of Pharmacy, New Mansoura University, New Mansoura, 7723730 Egypt

**Keywords:** Donepezil HCl, Pentoxifylline, Iso-absorptive point, Dual wavelength, Ratio subtraction, Ethoniosomes

## Abstract

Ethoniosomes have garnered significant interest as a novel class of lipid-based nano-carriers. Derived from the integration of ethanol and non-ionic surfactants within their vesicular structures, ethoniosomes exhibit superior physicochemical stability, enhanced skin permeability, and the capacity to encapsulate both hydrophilic and lipophilic drugs. In this study, three quick, easy, affordable, and environmentally friendly spectrophotometric methodologies have been developed to simultaneously assess Donepezil HCl (DPZ) and pentoxifylline (PTX) in their pure form, synthetic mixtures and laboratory-prepared ethoniosome formulation with a remarkable degree of precision and accuracy. The iso-absorptive point, dual wavelength, and ratio subtraction methods are the three approaches that have been successfully developed and validated. For both DPZ and PTX, the concentrations were rectilinear over the range 1–100 µg/mL. Validation of the suggested methods has been carried out in accordance with ICH standards with regard to linearity, detection limit, quantification limit, precision, and accuracy. The evaluation of whiteness, blueness, and greenness measures confirmed that the recommended strategies show a significant advantage over previously published ones.

**Supplementary Information:**

The online version contains supplementary material available at 10.1186/s13065-026-01759-4.

## Introduction

Central nervous system (CNS) disorders, including neurodegenerative diseases like Alzheimer’s disease (AD) and psychiatric conditions, present significant therapeutic challenges due to the complexity of brain physiology and the presence of the blood-brain barrier (BBB). The BBB serves as a protective mechanism, restricting the passage of most hydrophilic and high-molecular-weight drugs, thereby limiting the effectiveness of conventional drug delivery systems [1]. Despite advances in pharmacotherapy, the majority of CNS-active drugs suffer from poor bioavailability, rapid metabolism, and systemic side effects, highlighting the need for innovative drug delivery approaches.

The term “dementia” refers to a variety of symptoms that impair thinking, memory, and social skills in such a way which disrupt the daily activities of those who suffer from it. There is more than one type of dementia, which are caused by several disorders [2]. There are over 200 subcategories of dementia, but the most prevalent are Alzheimer’s (AD), vascular dementia (VaD), Lewy body, and mixed dementia (which frequently combines AD and VaD) [3]. The most prevalent cause of dementia is AD, which is a silent thief that progressively steals memory, cognition, and identity. It is a progressive neurodegenerative disorder associated with gradual cognitive deterioration severe enough to interfere with daily activities. The etiology of AD is suggested to be due to deposition of beta-amyloid proteins and neurofibrillary tangles in the brain interfering with normal cell function [4]. Additionally, the deficiency in acetylcholine neurotransmitter is another supposed cause of AD [5]. Despite extensive research, current pharmacological treatments for AD, including cholinesterase inhibitors (e.g., donepezil, rivastigmine) and N-methyl-D-aspartate (NMDA) receptor antagonists (e.g., memantine), offer only symptomatic relief and do not halt disease progression [6].

The second most common cause of dementia after AD is cognitive impairment due to a vascular disorder named as vascular dementia (VaD). Smoking, high blood pressure, high cholesterol, and stroke are among the factors that boost the risk of vascular disorders and subsequently raise the risk of VaD [7]. Patients with vascular diseases who may already have AD experience a more deteriorating cognitive state. Recent research has revealed that cholinergic transfer dysfunction in VaD patients is comparable to that observed in AD patients, suggesting that acetylcholinesterase inhibitors like DPZ may be beneficial for VaD patients [8].

**Fig. 1:**
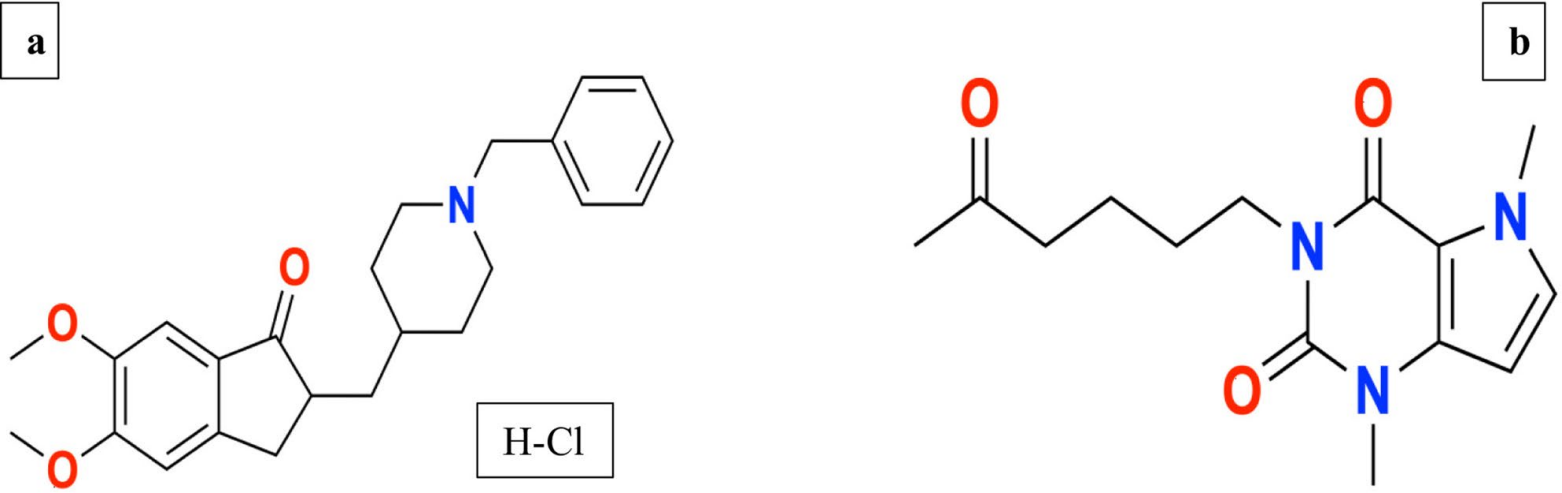
Chemical structures of (**a**) donepezil HCl (DPZ) and (**b**) pentoxifylline (PTX)

In this study, two of the most widely used medications for treatment of AD and VaD were analyzed by the developed spectrophotometric methods in their pure form and laboratory-prepared nanoformulation. The first drug is donepezil HCl (DPZ);2-[(1-benzylpiperidin-4-yl)methyl]-5,6-dimethoxy-2,3-dihydroinden-1-one; hydrochloride; Fig. 1a [9], , it is a white to off-white crystalline powder that is odorless and highly soluble in cloroform and water. It is barely soluble in ethanol and glacial acetic acid, insoluble in n-hexane, and only weakly soluble in acetonitrile and ethyl acetate [10]. The enzyme acetylcholinesterase, which typically breaks down acetylcholine, is selectively and reversibly inhibited by DPZ, which improves cholinergic transmission and alleviates AD dementia symptoms [11]. The official USP 38 monograph applied an HPLC-UV approach to quantify DPZ in sample and standard solutions at 274 nm using a mobile phase comprised of buffer adjusted at pH 1.8 utilizing perchloric acid and acetonitrile in the ratio (65:35) [12]. Many analytical approaches have been reported for quantification of DPZ in pure form, pharmaceutical dosage forms, or biological fluids involving spectrophotometric [13], spectrofluorimetric [14, 15] and HPLC [16] methodologies.

The second drug is pentoxifylline (PTX); (3,7-dimethyl-1-(5-oxohexyl)-3,7-dihydro-1 H-purine-2,6-dione) or 1-(5-oxohexyl)-3,7-­dimethylxanthine, Fig. 1b [17], , which is a methyl xanthine derivative that is freely soluble in chloroform and methanol, soluble in water, slightly soluble in ether and sparingly soluble in ethanol and toluene [18]. It functions as a nonselective phosphodiesterase inhibitor, capable of efficiently and rapidly traversing the blood-brain barrier upon oral administration. On the other hand, its oral bioavailability is only 20–30% due to high first-pass clearance [19]. It is a vasoactive agent that improves blood flow by reducing blood viscosity via enhancing erythrocyte flexibility, minimizing plasma fibrinogen, limiting neutrophil activation, and suppressing erythrocyte/platelet aggregation. It also possesses antioxidant and anti-inflammatory properties [20]. For the determination of PTX either alone or in combinations, many analytical approaches were reported involving spectrophotometric [21, 22] and chromatographic methods [23]. It was proven that PTX can be used as an add-on treatment with DPZ for AD and VaD, as it suppresses cell death, promotes blood flow, and has an anti-inflammatory impact, which alleviates cognitive issues brought on by cerebral ischemia [24].

Although traditional oral medications such as tablets and capsules have high oral bioavailability, their effectiveness is greatly diminished due to hepatic first-pass metabolism, plasma protein binding, and systemic side effects [25]. It has been proven that nano-carriers as a novel drug delivery system function as a barrier enclosing drug molecules in their inner core, shielding them from chemical or metabolic degradation in the exterior environment. Additionally, nano-carriers can enhance medication penetration via biological membranes. Niosomes and ethoniosomes have emerged as appealing vesicular carriers because of their capacity to encapsulate both hydrophilic and lipophilic medications, improve drug penetration, and increase therapeutic effectiveness [26]. Niosomes are vesicular systems composed of cholesterol and non-ionic surfactants, forming bilayer structures similar to liposomes but with higher chemical stability and cost-effectiveness [27]. These vesicles have several advantages, including controlled drug release, enhanced permeability, and conservation of encapsulated medicines from degradation. Niosomes have been extensively investigated for transdermal, oral, and intravenous drug delivery, especially in cancer therapy, antimicrobial therapies, and CNS drug delivery [28]. Ethoniosomes are an improved form of niosomes, including ethanol in their composition to boost drugs’ flexibility and penetration. By disrupting the lipid bilayer, ethanol promotes membrane fluidity and makes it possible to penetrate more deeply through biological barriers, especially the skin and blood-brain barrier. This distinctive feature renders ethoniosomes highly valuable for transdermal and intranasal drug delivery, permitting the non-invasive administration of CNS medications while avoiding hepatic first-pass metabolism and minimizing systemic adverse effects [29].

To the extent of our knowledge, only one reported HPLC-MS/MS method was developed and validated for simultaneous determination of the co-administered mixture of DPZ and PTX [30]. The reported method has utilized an Imtakt Cadenza^®^ CD-C18 (100 × 3 mm, 3 μm) column for chromatographic separation, a mobile phase composed of methanol and 0.1% formic acid in water (80:20, v/v) and a 0.2 mL/min flow rate. HPLC-MS /MS is a highly sensitive and selective technique of analysis that can separate and analyze a multicomponent sample. However, it is a costly technique of analysis that requires an expert staff to operate and as a result, it is not readily available in all quality control labs [31]. In contrast to other chromatographic methods of analysis, chemometrics offers green, rapid, reliable, and inexpensive methods for resolving challenging mixtures with overlapping spectra without the need for pre-analysis separation [32]. Both academic and quality control laboratories may readily use them without requiring costly equipment, particular software, or training. This encouraged us to develop the first green, very simple, and affordable spectrophotometric chemometric methods which are sensitive enough for analysis of DPZ and PTX in their pure form and laboratory co-formulated ethoniosomes. The developed approaches have been validated for their greenness using two assessment tools: the Analytical GREEnness metric (AGREE) [33] and the Modified Green Analytical Procedure Index (MoGAPI) [34]. It is vital for QC laboratories to implement practices that meet the standards needed for efficacy and feasibility in addition to being ecologically friendly. Therefore, the RGB algorithm was employed to evaluate the whiteness of the analytical procedure [35]. Furthermore, in accordance with the fundamental principles of sustainable environmental practices, contemporary concepts of blueness have been adopted [36].

## Experimental

### Apparatus

The spectrophotometric measurements were conducted using a Shimadzu UV-Visible double-beam spectrophotometer (Kyoto, Japan). It was equipped with matching quartz cuvettes with a 1 cm path length at a 200–800 nm range. UV-Probe software (version 2.1) was employed to manage the instrument’s operations.

### Chemicals and solvents

All utilized materials and reagents through this work are of analytical grade. Pure DPZ standard powder (99.8%) was given as a gift from HIKMA PHARMA, Cairo, Egypt. Pure PTX powder was received as a gift from SANOFI AVENTIS, Cairo, Egypt. HPLC-grade methanol and ethanol were purchased from Sigma-Aldrich Co. (Steinheim am Albuch, Germany). Cholesterol (5-cholesten-3beta-ol) was obtained from Oxford fine chem lab. Span 60 was purshased from >>>>. Freshly prepared double-distilled water was employed for carrying out the experiments.

### Preparation of the co-formulated ethoniosomes

**Fig. 2:**
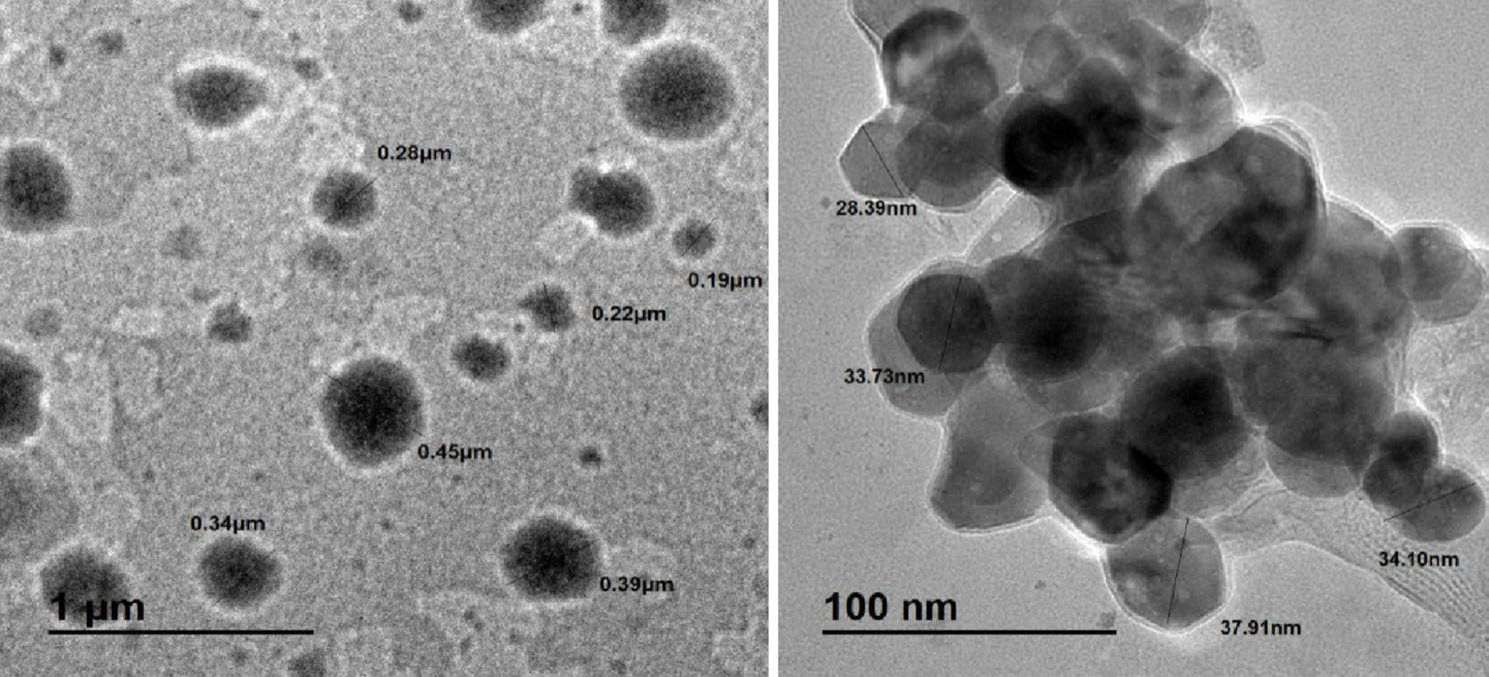
Transmission electron Microscope images of the laboratory prepared nano-sized ethoniosomes

Ethoniosomes have been generated using the ethanol injection approach as reported in a previous study with minor modifications [37]. The first step involved dissolving a specified quantity of surfactant/lipid mixture of Span 60 and cholesterol in equimolar ratio (5:5) in 3 mL of ethanol and injecting them into a warmed aqueous phase that was maintained at 60 °C which contained 12 mL of DPZ (1 mg/mL) and 12 mL of PTX (1 mg/mL) in a hydroalcoholic solution of 20% ethanol and 80% phosphate buffer (pH 7.4) v/v. The mixture was then stirred with a magnetic stirrer (Barnstead Thermolyne, SPA1020B, USA) for a further half an hour at room temperature. A water-bath sonicator (Branson, ESS-1, Mexico) was utilized for further size reduction of the generated ethoniosomes. As seen in Fig. 2, a microscopical inspection was performed to verify the development of ethoniosomes nano-sized vesicles.

### Preparation of standard stock solutions

Freshly prepared double-distilled water has been employed as the preferable green solvent owing to the free solubility of DPZ and PTX in water [38, 39]. After being accurately weighed, 10 mg of each of DPZ and PTX were transferred into two separate 100 mL measuring flasks. The stated drugs were then dissolved in 30 mL of the solvent, and then sonication for 10 min was done. The volume was subsequently raised up to 100 mL, yielding a standard stock solution with a concentration of 100 µg/mL. The solutions were kept in the refrigerator until needed.

### General methods of analysis

#### Construction of the calibration curves

**Fig. 3:**
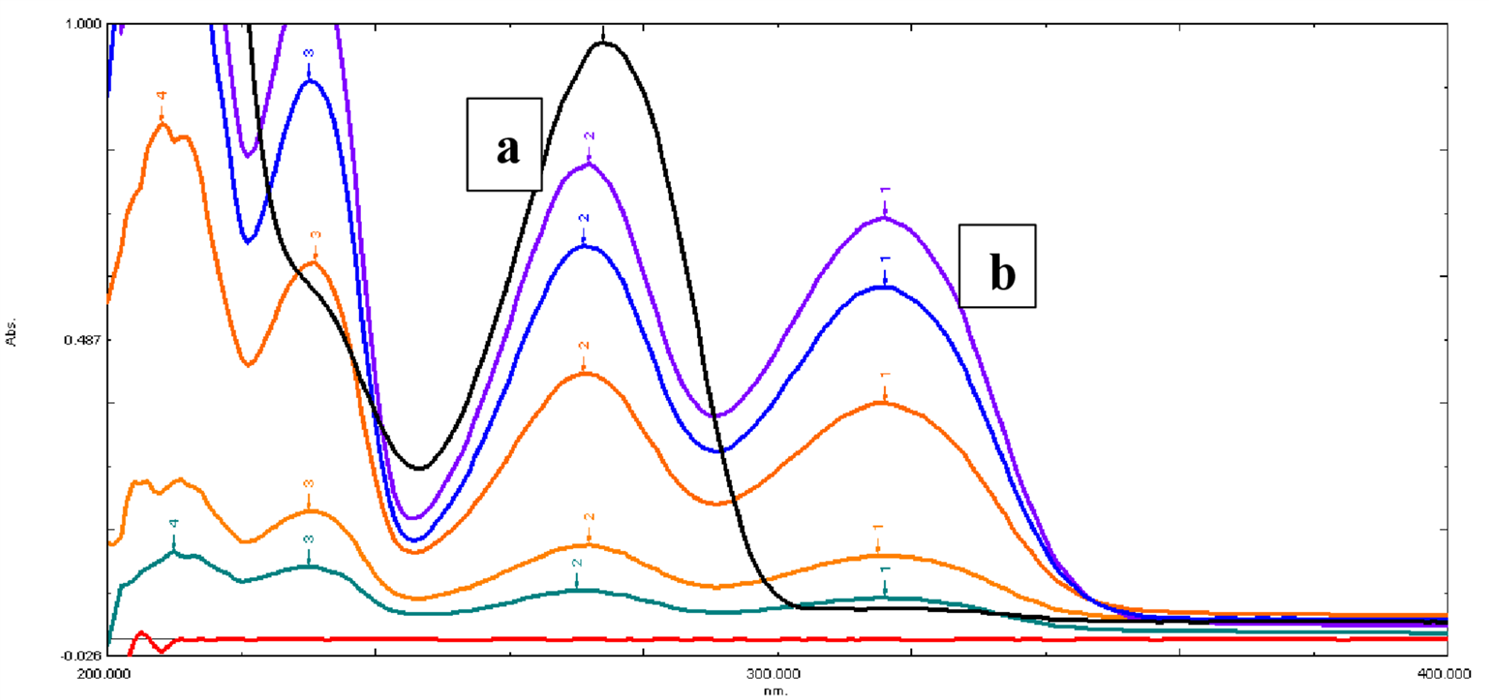
The overlaid zero-absorption spectra of (**a**) PTX and (**b**) DPZ showing maximum wavelengths at 273 and 316 nm for both drugs, respectively

Distilled water has been used as a blank to record the zero-order absorption spectra of the cited drugs over the range 200–600 nm. To achieve a final drug concentration of 1–100 µg/mL for both DPZ and PTX, accurate volumes from the stock solutions were measured and placed into a series of 10 mL volumetric flasks, diluted with the same solvent, and properly mixed. The calibration curves were constructed by plotting the absorbances of the serial dilutions of the cited drugs versus their final drug concentrations at λ 316 nm and 273 nm for DPZ and PTX, respectively, and the corresponding regression equations were then generated. DPZ has been determined directly at λ 316 nm, where PTX has zero interfering absorbance, as stated under “Construction of the calibration curves”, as shown in Fig. 3.

#### Method I: Iso-absorptive point method [40]

**Fig. 4:**
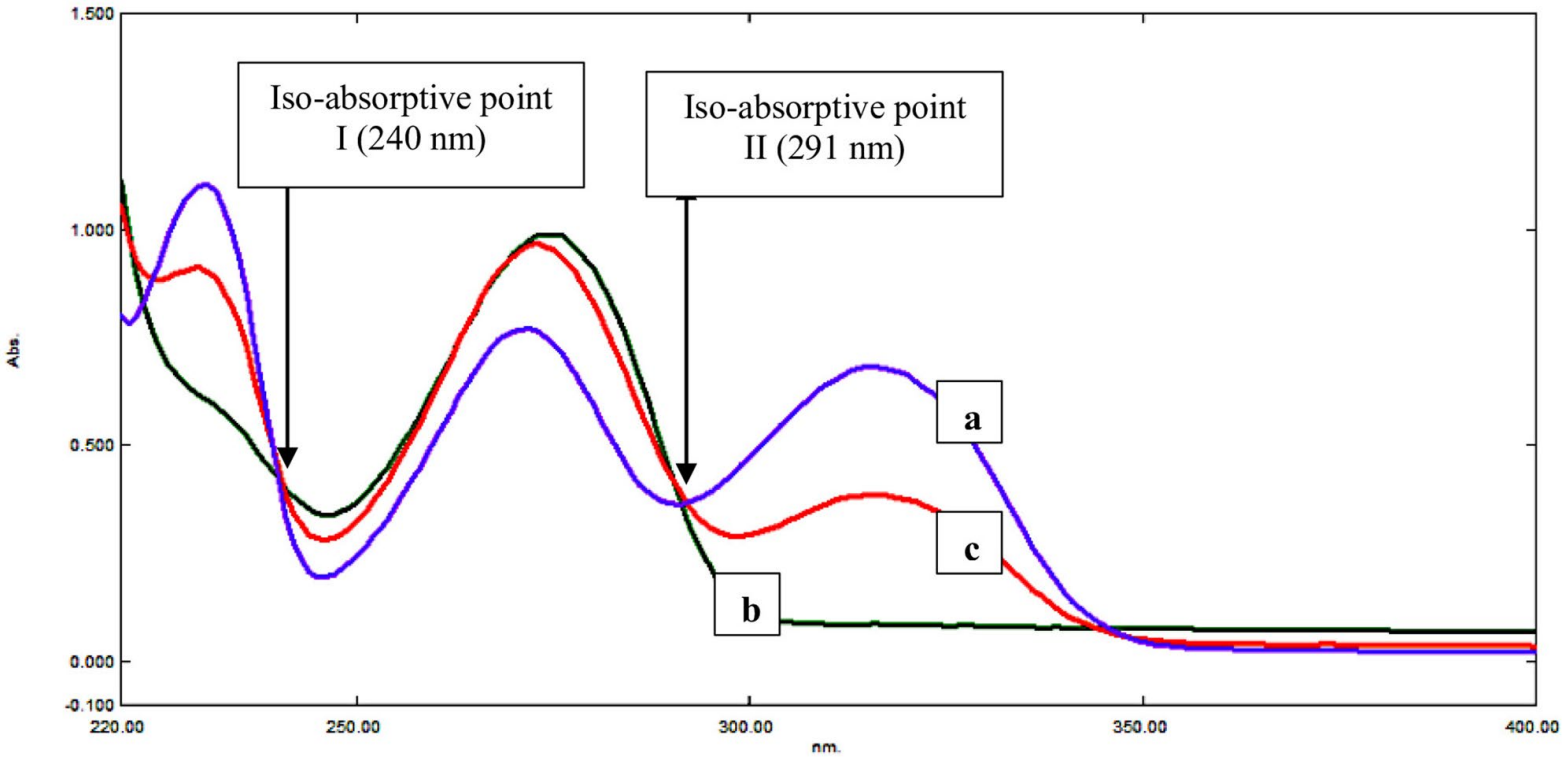
Zero-order absorptin spectra with two points of intersection of (**a**) DPZ (100 µg/mL), **b** PTX (100 µg/mL) and **c** a mixture containing 50 µg/mL of each drug revealed that 291 nm is an iso-absorptive point and that PTX has no absorbance at 316 nm

An equimolar concentration solution for the two drugs shows the same/equal absorbance and absorptivity at a certain wavelength, which is named the iso-sbestic point or iso-absorptive point (ISP). If there is a mixture of the two drugs, we may use the mixture’s absorbance at ISP to calculate the total concentration of both medications. The concentration of one of them must be determined via a different technique, and then the concentration of the other can be calculated by subtraction. A calibration curve for the zero-order absorbance of DPZ can be directly constructed at λ 316 nm versus its concentration in µg/mL, where PTX shows a plateau region with no absorbance at this wavelength. For PTX quantification, DPZ (100 µg/mL), PTX (100 µg/mL), and a mixture of equal amounts of the two medications (50 µg/mL DPZ and 50 µg/mL PTX) were all evaluated for the development of their zero-order absorption spectra at the ISP at 291 nm, as illustrated in Fig. 4. A calibration curve was constructed for the zero-order absorption of DPZ or PTX at the ISP against their corresponding concentrations (1–100 µg/mL) and the regression equation was estimated. The total drug concentration in the mixture was calculated using the linear regression equation derived from the calibration curve at 291 nm. DPZ concentration can be determined from the mixture’s estimated regression equation at 316 nm, and then PTX concentration is then calculated by subtraction from the total mixture concentration at 291 nm. This method’s fundamental weakness is that it necessitates a plateau region where one of the two drugs shows no absorbance or a different analytical approach is available in order to determine one of the mixture’s analytes.

#### Method II: dual wavelength method [41–43]

In order to employ this method, the overlapped spectra must have two wavelengths, at which one of the two drugs, named interfering drug B, exhibits equal absorbance at both wavelengths (∆Abs._B_ = zero), while the other drug of interest (A) displays a crucial variation in absorbance (∆Abs._A_) between the two wavelengths, which is directly related to its concentration. This enables the determination of drug A, whereas B may be determined immediately from the plateau region of the spectrum lacking A absorbance. For the PTX calibration curve, the difference in absorbance of its solutions at 277 nm and 316 nm was recorded versus their corresponding concentrations over the range (1–100 µg/mL). ΔAbs._PTX_ was calculated as [Abs._PTX_ at 277 nm – Abs. _PTX_ at 316 nm] and plotted against their corresponding concentrations and PTX concentration can be estimated from the generated regression equation.

**Fig. 5:**
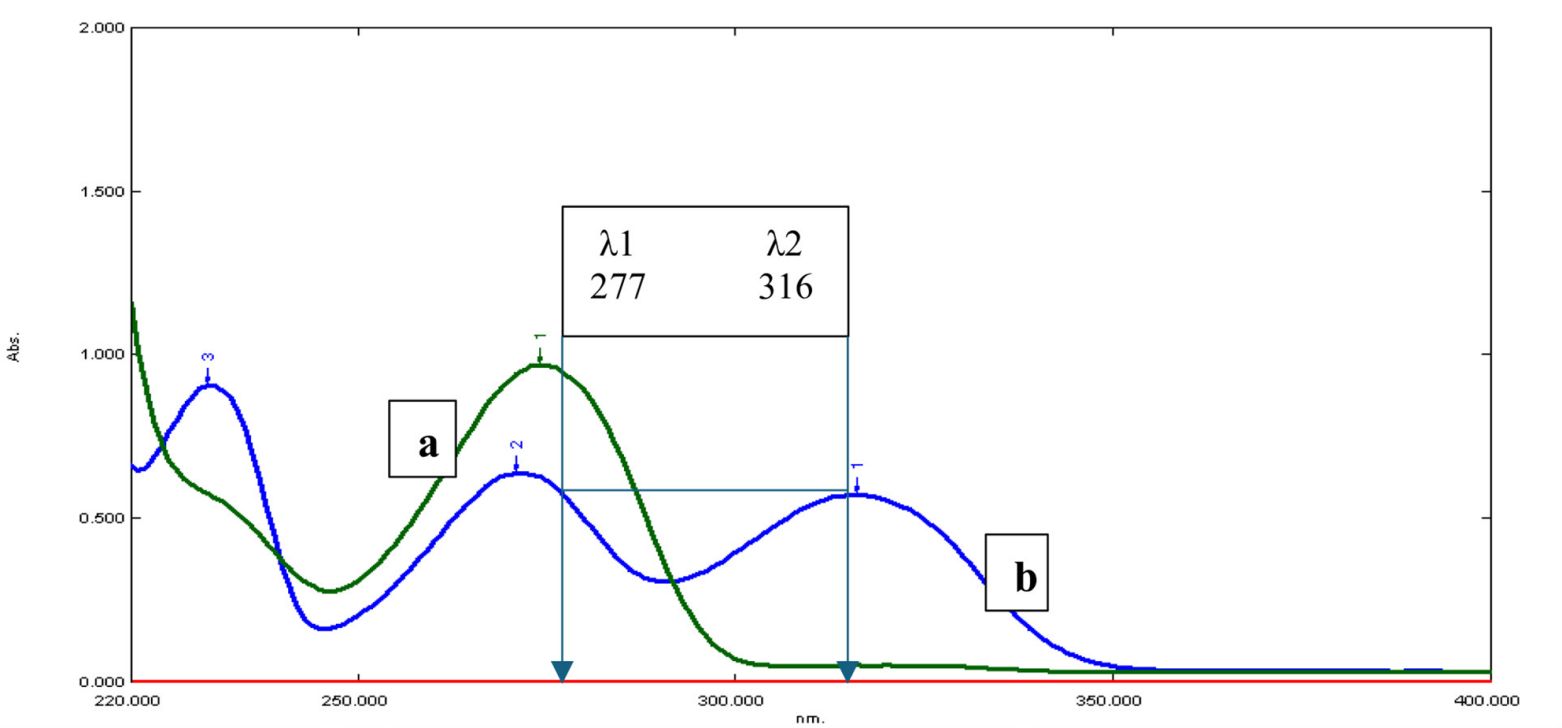
Zero-order spectra of (**a**) PTX 100 µg /mL and (**b**) DPZ 100 µg /mL showing the two selected wavelengths (277 and 316 nm) for the dual-wavelength approach

#### Method III: ratio Subtraction method [44]

This approach is effective for determining two combined medications (A and B) with overlapping spectra as long as one of them (B) has a zero-order prolonged region in its spectrum where A has no interfering absorbance. To calculate the concentration of medication A, using the ratio subtraction method, divide the mixture’s zero-order spectrum by the zero-order spectrum of a certain concentration of B, known as the divisor (B’). This causes a steady absorbance in the extended portion of the drug (B).

$$ \left( {{\mathrm{A}}{\mkern 1mu} + {\mkern 1mu} {\mathrm{B}}} \right)/\left( {{\mathrm{B}^{\prime}}} \right){\text{ }} = {\text{ }}\left( {{\mathrm{A}}/{\mathrm{B}^{\prime}}} \right){\text{ }} + {\text{ constant}}$$

 

The original spectrum of A may be separated by subtracting this constant and multiplying the resultant spectra by the divisor (B’). The linear regression equation of drug A calibration curve at its λmax can then be used to calculate its concentration. Drug B may be directly quantified using the absorbance at the wavelength in the spectrum where drug A is not showing any absorption. A calibration curve has been generated for PTX (A) at its λmax at 273 nm, utilizing a range of its standard solutions (1–100 µg /mL). A 50 µg/mL of DPZ (B) was chosen as the divisor (B’), and its spectra were recorded. The spectrum of a binary mixture of PTX and DPZ (A + B) was recorded and divided by the divisor (B’) so a novel ratio spectrum was generated. In the plateau area (300–350 nm), PTX (A) has no absorbance so the division produced constant absorbance values equal to (B / B’) throughout this region. The fixed absorbance ratio (B / B’) was subtracted from the ratio spectrum ([A + B] / B’) to yield a new spectrum (A/B’). By multiplying the resulting spectrum by B’, the original PTX spectrum was resolved and its concentration can be easily determined from the calibration curve’s generated regression equation.

**Fig. 6:**
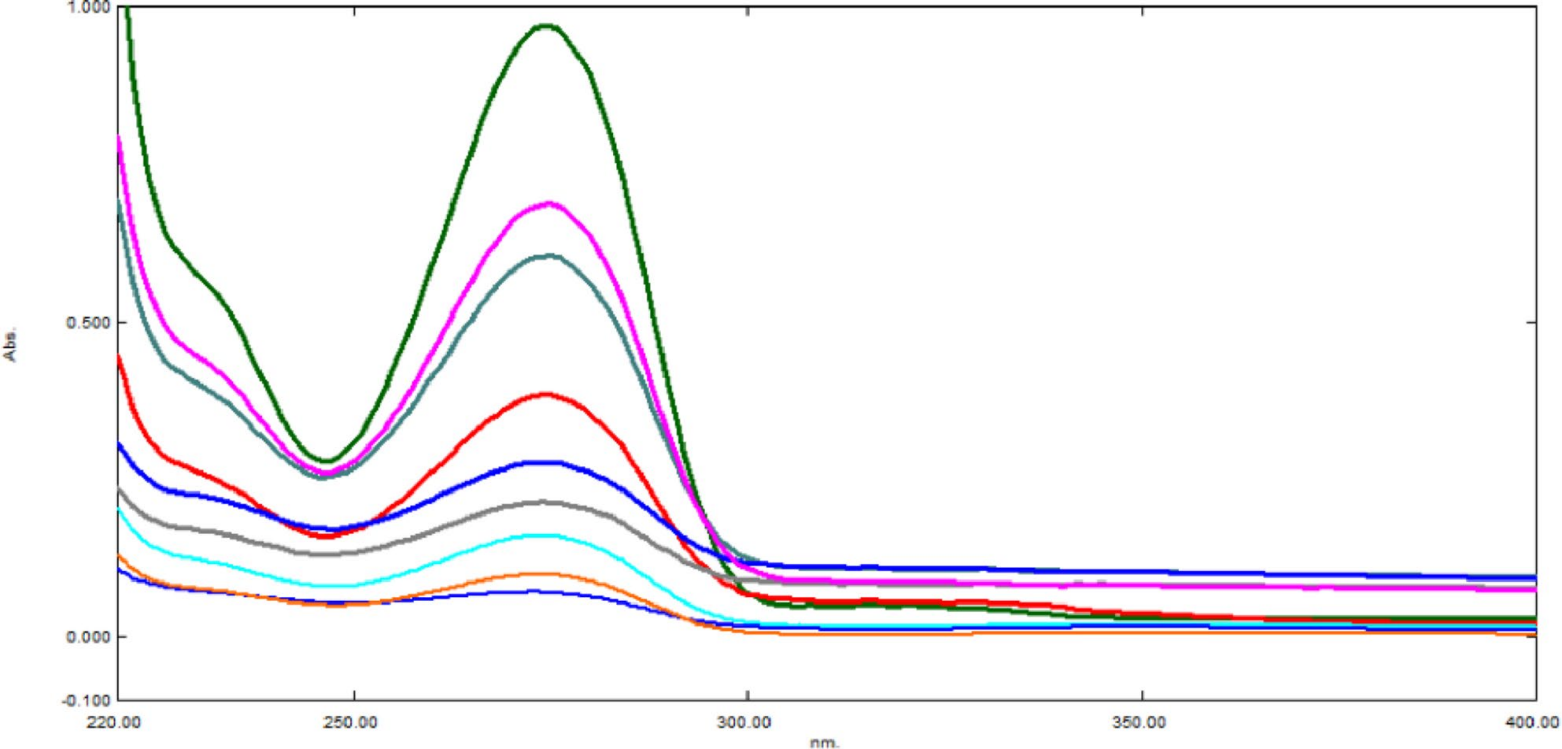
The ratio subtraction approach was implemented to resolve the spectra of PTX in the combination at its λmax 273 nm at various concentrations (1–100 µg/mL)

### Analysis of laboratory‑prepared mixtures

Precise volumes of DPZ and PTX stock solutions were transferred to a set of 10 mL measuring flasks in different ratios (1:1, 1:5, 5:1, 1:10 and 10:1). Mixtures have been generated and diluted to the mark using distilled water as the green solvent of choice. Both cited drugs have been determined in their laboratory-prepared mixtures using the aforementioned spectrophotometric methodologies employing the procedure outlined under “Construction of the calibration curves”.

### Analysis of the laboratory-formulated ethoniosomes

The medications under study have been extracted from the laboratory-manufactured ethoniosomes utilizing methanol in a single-step extraction method. Each milliliter of the formulation involves 1 milligram of both DPZ and PTX. A 100 µg/mL solution was obtained by transferring 1 mLof the nano-sized formulation to a 10 mL volumetric flask, then the same solvent was used to dilute it up to the final volume. After 5 min of sonication, the solution was further diluted to achieve concentrations within the specified linearity ranges. The corresponding concentrations were subsequently estimated by applying the procedures mentioned below in “Construction of the calibration curves”.

## Results and discussion

Novel UV spectrophotometric approaches are mostly applied for multicomponent analysis, limiting the laborious extraction processes of interferents and allowing the detection of an increasing number of analytes, hence minimizing analysis time and expenses. Multicomponent UV spectrophotometric strategies rely on recording and statistically analyzing absorption spectra [45]. The co-administered mixture of DPZ and PTX has shown an overlapped spectrophotometric absorption throughout the PTX absorption spectrum ranging from 240 to 300 nm, while DPZ can be directly determined in the presence of the non-interfering PTX. In the current study, three smart methods have been developed and validated for simultaneous analysis of the cited drugs spectrophotometrically for the first time with no need for prior extraction processes or physical separation in their pure form, synthetic mixed mixtures, and laboratory-prepared ethoniosomes. Chemometric approaches have been selected for their reliability, convenience of use, speed, performed, thus rendering them acceptable for routine analysis.

### Method optimization

#### Iso-absorptive point method

The absorption spectra of equivalent amounts of DPZ and PTX overlap between 240 and 300 nm, intersecting at two wavelengths (240 and 291 nm). Following a thorough assessment, 291 nm has been selected for superior accuracy based on the estimated % recoveries. To verify the iso-absorptive point, absorbance was measured for 100 µg/mL DPZ, 100 µg/mL PTX, and a combination of 50 µg/mL DPZ and 50 µg/mL PTX as illustrated in Fig. 4. DPZ was directly determined in the mixture at its λmax at 316 nm, while PTX was determined by subtraction from the total mixture concentration at 291 nm. With correlation coefficients ≥ 0.999, the calibration curves revealed conventional linear correlations between concentrations and zero-order absorbancesin the range of 1–100 µg/mL for both medications.

### Dual wavelength method

Following a careful examination of the DPZ and PTX spectra, the wavelengths 277 nm and 316 nm were determined to be the most suitable for this technique. DPZ absorbance was the same at both wavelengths (ΔAbs. DPZ = zero), but PTX absorbance varied significantly, and this fluctuation was directly related to PTX concentration. The following stage was to create a linear calibration graph using PTX concentrations and the associated absorbance variations at both wavelengths (A277 nm and A316 nm). By using this calibration graph, the concentration of PTX in the mixture was directly determined based on the estimated regression equation. Using the mixture’s absorbance at 316 nm, the concentration of DPZ could be determined directly where PTX has no interfering absorbance (Fig. 5).

### Ratio Subtraction method

The spectrum of the mixture was recorded over the range 200–400 nm and then divided by the spectrum of 50 µg/mL DPZ as the divisor (B`). The spectrum that was generated (Spectrum 1) corresponds to [(PTX/DPZ) + constant]. The ratio spectrum of PTX/DPZ (Spectrum 2) was revealed after deducting the constant, the latter represents the absorbance plateau area between 310 and 330 nm. The PTX original spectrum was resolved by simply multiplying the ratio spectrum (Spectrum 2) by B`. Currently, PTX can be determined using the established calibration graphs from the resolved spectrum at its λmax at 273 nm, as demonstrated in Fig. 6. DPZ can be directly estimated from the mixture’s absorption spectrum at its λmax of 316 nm, where PTX exhibits no absorbance.

### Validation of the suggested methods

The International Council for Harmonization’s ICH Q2 (R2) specifications have been employed to assess the linearity, accuracy, precision, and robustness of the proposed approaches [46].

### Linearity and concentration ranges

The calibration curve of DPZ was developed by plotting the absorbance readings versus their matching concentrations at 316 nm and a linear relationship over the range 1 to 100 µg/mL was observed, as indicated by the outstanding correlation coefficient value (r) in Table 1. The values of LOD and LOQ for every single drug have been calculated using the following specified equations, in compliance with ICH guidelines [46].

LOD = 3.3 × (σ/S) and LOQ = 10 × (σ/S), where σ is the standard deviation of the response and S is the slope of the standard calibration curve.

The LOD and LOQ values of DPZ were found to be 0.177 and 0.537 µg/mL, respectively. For method I, PTX was manipulated in the same way but at the iso-absorptive point of 291 nm. Table 1 lists the elements from the linear regression equation, showing a correlation coefficient of 0.999 throughout a linear range of 1 to 100 µg/mL, with a LOD of 0.32 µg/mL and LOQ of 0.96 µg/mL.

For method II, regression analysis revealed an acceptable linear correlation (*r* = 0.999) between PTX ΔAbs. values (Abs. 277 nm – Abs. 316 nm) and their respective concentrations throughout a range of 1–100 µg/mL with a LOD of 0.361 µg/mL and LOQ of 1.095 µg/mL, as indicated in Table 1. For method III, the calibration graph and linear regression equation have been generated using the PTX resolved absorption spectrum with its λmax at 273 nm. Over a concentration range of 1–100 µg/mL (Table 1), a correlation coefficient of 0.999 was obtained, with a corresponding LOD of 0.29 µg/mL and LOQ of 0.88 µg/mL.

**Table 1:** Analytical performance data for analysis of DPZ and PTX by the suggested spectrophotometric methodologies

Parameter	DPZ	PTX		
	Direct	Method I	Method II	Method III
Wavelength (nm)	316 nm	291 nm	277–316	273 nm
Linearity range (µg/mL)	1–100	1–100	1–100	1–100
Intercept (a)	0.0763	0.1116	0.1505	0.2508
Slope (b)	0.0061	0.0026	0.0090	0.0075
Correlation coefficient (r)	0.9999	0.9999	0.9999	0.999
%RSD	1.01	1.00	1.003	0.998
%Error	0.408	0.408	0.354	0.408
LOD (µg/mL)	0.177	0.316	0.361	0.291
LOQ (µg/mL)	0.537	0.958	1.095	0.881
S_a_	0.0003	0.0002	0.001	0.0007
S_b_	0.0000	0.0000	0.0000	0.0000
S_y/x_	0.0005	0.0005	0.0021	0.0011

### Accuracy

Accuracy is defined as how closely the test results match their real or theoretical values [46]. To evaluate the accuracy of the suggested approaches, laboratory-prepared mixtures of DPZ and PTX at various known concentrations were employed. The novel methods were applied for each mixture, and the absorbance values have been recorded and utilized to determine the concentrations of the relevant medications in the associated linear regression equations. Table 2 indicates the accuracy of the suggested methodologies based on the substantial mean % recoveries and low %RSD values obtained from comparing the percent of the concentrations estimated using the appropriate regression equations to the known values.

### Precision

The degree of agreement among several measurements performed on the same substance under similar circumstances is defined as an analytical method’s precision [46]. The recommended approaches have been applied in three replicates on three DPZ and PTX various combinations prepared in the lab. An intra-day analysis (within-day precision) was conducted on the same day at three distinct times, and an inter-day analysis (between-day precision) was conducted within three consecutive days. As illustrated in Table 3, the relatively small values of percentage relative standard deviation (%RSD) reflect high consistency and confirm the remarkable precision of the established approaches.

**Table 2:** Analysis of DPZ and PTX in laboratory-prepared mixtures using various suggested methodologies

Mixture	Ratio	%Recovery^∗^			
		DPZ	PTX		
		Direct (316 nm)	Method I	Method II	Method III
1	1:1	101.05	100	98.95	97.86
2	1:5	98.25	98.55	99.22	98.00
3	5:1	100	102.1	101.05	98.4
4	1:10	98.68	99.03	100.66	98.66
5	10:1	100	100.1	99.37	99.96
Mean		99.59%	99.96%	99.85%	98.58%
%RSD		1.13	1.37	0.94	0.84

^∗^The value is the average of three distinct determinations

### Application of the proposed methods

#### Assay of the laboratory prepared ethoniosome nanoformulation

The approaches proposed in this study have been applied for quantification of DPZ and PTX in the laboratory co-formulated ethoniosomes. Only one step of methanol extraction was carried out for sample processing, revealing the removal of all interfering excipients. This highlights the practicability of the suggested methods as an environmentally conscious option for assessing the quality of analytes. The recovery data from the proposed methods have been statistically compared to those obtained from the reference methods [16, 47] using the Student’s t-test and variance ratio F-test [48]. As apparent in Table 4, there have been no statistically substantial variations between the recoveries obtained by the suggested techniques and those gained from the previously reported procedures.

**Table 3:** Precision data for the intra-day and inter-day analysis of DPZ and PTX by the suggested approaches

Concentration(µg/mL)		% Recovery^∗^ ± RSD			
		Within-day Precision		Between-day Precision	
DPZ	PTX	DPZ^#^	PTX	DPZ^#^	PTX
Method I: Iso-absorptive point method					
50	50	100.17 ± 1.60	100.25 ± 1.14	99.96 ± 1.16	99.52 ± 1.47
10	50	99.44 ± 1.35	100.10 ± 1.65	99.13 ± 1.25	99.50 ± 1.59
50	10	99.00 ± 1.01	100.54 ± 1.74	100.31 ± 1.15	100.08 ± 1.69
Method II: Dual wavelength method					
50	50	99.74 ± 0.86	100.79 ± 0.11	100.42 ± 0.80	99.80 ± 0.96
10	50	98.28 ± 0.35	98.23 ± 0.37	101.57 ± 0.42	99.33 ± 0.71
50	10	99.97 ± 0.84	100.01 ± 1.13	100.23 ± 0.33	100.52 ± 0.88
Method III: Ratio subtraction method					
50	50	99.87 ± 1.05	99.95 ± 1.26	100.85 ± 1.18	100.01 ± 1.38
10	50	99.80 ± 1.66	100.78 ± 1.57	99.28 ± 1.72	100.24 ± 1.31
50	10	100.18 ± 1.29	100.01 ± 1.38	100.18 ± 1.79	100.23 ± 1.93

^∗^The value is the average of three distinct determinations

^#^DPZ has been determined directly using its absorbance at 316 nm

**Table 4:** Quantification of DPZ and PTX in their laboratory co-formulated ethoniosomes using the proposed methods and comparison with the reference methods

Method	% Recovery^a^±RSD	
	PTX	DPZ^c^
Method I (Iso-absorptive method)	100.34 ± 0.99	100.01 ± 1.38 (t = 0.187, f = 1.359)
	(t = 0.620, f = 1.160)	
Method II (Dual wavelength method)	99.76 ± 1.01	
	(t = 0.342, f = 2.574 )	
Method III (Ratio subtraction method)	100.16 ± 0.99	
	(t = 0.021, f = 1.672)	
Reference methods [47, 48]	100.17 ± 1.68	100.18 ± 1.79

^a^The value represents the mean of six readings for both the suggested and reported approaches

^b^The t-value and F-value are the values included in parenthesis. At 95% confidence level, the tabulated values are t = 2.228 and F = 5.053

^c^DPZ was directly quantified by measuring the absorbance at λ 316 nm

### Statistical analysis

The findings obtained from implementing each of the suggested approaches and the published methods [16, 47] were compared using Student’s t-test and variance ratio F-test [48] to assess how effectively the suggested methods can be utilized in determining DPZ and PTX in pure powder. According to Table 5, the comparison showed no noteworthy differences at the 95% confidence level.

**Table 5:** Analysis of the cited drugs in laboratory prepared synthetic mixtures using both the suggested and reported approaches

Method	% Recovery^a^±RSD	
	PTX	DPZ^c^
Method I (Iso-absorptive method)	99.97 ± 1.85	99.64 ± 1.41 (t = 0.182, f = 1.050)
	(t = 0.323, f = 1.608)	
Method II (Dual wavelength method)	100.18 ± 1.68	
	(t = 0.569, f = 1.335)	
Method III (Ratio subtraction method)	100.13 ± 0.95	
	(t = 0.661, f = 2.327)	
Reference methods [47, 48]	99.66 ± 1.46	99.49 ± 1.45

^a^The value represents the mean of six readings for both the suggested and reported approaches

^b^The t-value and F-value are the values included in parenthesis. At 95% confidence level, the tabulated values are t = 2.228 and F = 5.053

^c^DPZ was directly quantified by measuring the absorbance at λ 316 nm

### Assessment of the proposed methods’ greenness

In scientific research, quality assurance, and pharmaceutical development, analytical chemistry plays a crucial role. However, classical methods of analysis frequently contribute to resource depletion and adverse ecological effects by using potentially hazardous chemicals and solvents, consuming excessive amounts of energy, and generating plenty of waste. To tackle these issues, environmentally benign and practically reliable analytical procedures could be developed using sustainable strategies like Green Analytical Chemistry (GAC) [49] and White Analytical Chemistry (WAC) [50]. While greenness focuses on environmental impact, WAC promotes the analytical method’s balance between sustainability, robustness, and analytical quality. Assessing the greenness and whiteness of analytical procedures is vital for making sure that contemporary methods satisfy sustainability and analytical performance standards. Several eco-friendly spectrophotometric-based analytical methods have been developed to determine drugs either alone [51] or in combinations [52] applying various tools for greenness and whiteness assessment. The present study has employed different sustainability evaluation tools including AGREE, MOGAPI, BAGI, and the RGB Model.

#### I: analytical greenness metric (AGREE)

**Fig. 7:**
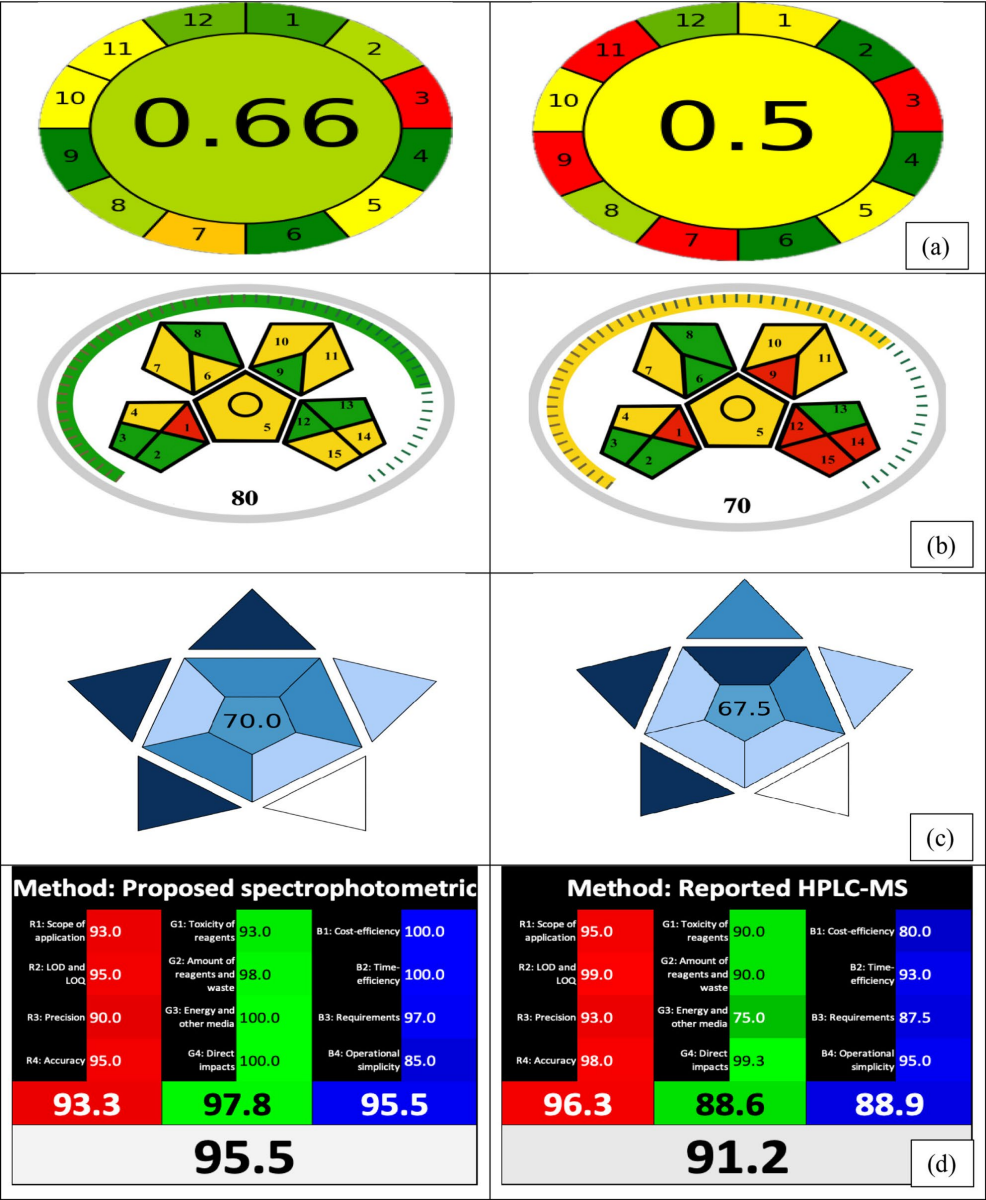
Evaluation of greenness and whiteness of the proposed method and previously reported method [30] using various tools **a** AGREE, **b** MoGAPI, **c** BAGI, and **d** RGB_12_ Model

Among the various assessment tools developed, the AGREE metric has gained significant recognition as a comprehensive and user-friendly tool for assessing the environmental sustainability of analytical methods [33]. It is a quantitative and visual evaluation tool designed to reflect the extent to which a given analytical procedure complies with the 12 Principles of GAC. Individual scores on a range from 0 to 1 are assigned for each principle, and the sum of these scores yields the ultimate greenness score, which is typically between 0 and 1. This final score will be displayed in the center of a circular, color-coded figure, which permits easy and quick interpretation. This visual representation not only identifies strengths and weaknesses within a technique, but it also facilitates the comparison with various procedures according to their ecological effect. The estimated AGREE score of 0.77 indicates that the developed approaches are ecologically friendly, as illustrated in Fig. 7(a).

#### II: modified green analytical procedure index (MoGAPI)

The Green Analytical Procedure Index (GAPI) [53] has emerged as a widely adopted metric for greenness assessment due to its simplicity and visual interpretability, from sample collection to final measurement. GAPI employs a pictogram with five pentagonal sections, each fragmented into segments that represent different steps of the analytical process: sample preparation, chemicals and solvents employed, instruments, and waste generation. Each section is color-coded (green, yellow, or red) to represent a low, medium, or significant environmental effect, accordingly. This semi-quantitative visual representation allows for fast comparison and identifies particular areas where an analytical process might be improved. Despite its extensive application, GAPI has some drawbacks. It frequently lacks clarity in areas like energy use, waste management methods, and instrument-specific sustainability.

Therefore, the Modified Green Analytical Procedure Index (MoGAPI) [34], a novel semi-quantitative framework, has been designed in 2024 to enhance the accuracy, inclusivity, and objectivity of greenness assessments. MoGAPI incorporates additional parameters such as instrument energy requirements, sample throughput, and waste management practices, while maintaining the intuitive visual presentation that made GAPI accessible. By employing MoGAPI, the chart’s overall score of 87 and the color of the scale surrounding the pentagrams indicate the method’s exceptional greenness, as illustrated in Fig. 7(b).

### Assessment of the proposed methods’ blueness

Blue Applicability Grade Index (BAGI) was launched as a novel measure for assessing the practicality of an analytical procedure. BAGI can be considered as a supplement to known green metrics, primarily concentrating on the “blue” concepts of WAC dealing with the practical elements [36]. BAGI evaluates ten criteria to provide a pictogram and a score that reflect the feasibility and effectiveness of an analytical method. A consecutive blue color scale was employed to illustrate the ultimate score, with distinct shades of dark blue, blue, light blue, and white to indicate high, medium, low, and inadequate compliance with the established standards, respectively. The overall score should exceed 60 for the analytical procedure to be deemed “practical”. The key benefit of BAGI is its ease and convenience of implementation, facilitated by an engaging online application that enables rapid evaluation of the approach and the generation of a colored pictogram illustrating the evaluation results. In order to ensure a comprehensive assessment, BAGI was employed, and our suggested approaches scored 77.5 on the BAGI assessment tool. Figure 7(c) illustrates the applicability and effectiveness of the recommended methodologies in reality.

### Assessment of the proposed methods’ whiteness

The Red–Green–Blue (RGB_12_) model [35], named for the primary colors of light–red, green, and blue, provides a thorough framework for assessing the “whiteness” of an analytical method, with “white” representing the optimal combining of analytical performance (red), ecological friendliness (green), and practical application (blue). The format has 12 criteria in a ready-to-use Excel sheet, with four designated to each color domain, offering a systematic paradigm for method assessment. The RGB12 model enables objective evaluation and enhances analytical techniques, ensuring they are scientifically reliable, ecologically sustainable, and practically effective. As seen in Fig. 7(d), our proposed methods show 92% whiteness, which emphasizes a balance between sustainability, analytical performance, and practicality.

## Conclusion

In summary, quick and straightforward spectrophotometric methods have been established to quantify DPZ and PTX concurrently, addressing the requirements of quality control laboratories. Those simple, cost-effective procedures may be favored over more expensive, complex alternatives for the regular testing of the analyzed drugs in the co-formulated dosage form without prior separation. DPZ could be determined without interference from PTX, while PTX determination in the mixture has been challenging due to significant spectrum overlap. Chemometric methodologies have been chosen for their capacity to resolve complicated mixtures utilizing a spectrophotometer and simple mathematical calculations. DPZ could be determined without interference from PTX, while PTX determination in the mixture has been challenging due to significant spectrum overlap. Chemometric methodologies have been chosen for their capacity to resolve complicated mixtures utilizing a spectrophotometer and simple mathematical calculations. The cited drugs in combination have been analyzed in pure powders and laboratory-prepared ethoniosome co-formulation, implementing iso-absorptive point, dual wavelength and ratio subtraction approaches. The procedures have been quick, simple, and ecologically benign, utilizing few solvents. The suggested approaches have been checked for routine inspection of analytes in laboratories of quality control, demonstrating sufficient reproducibility and accuracy.

## Supplementary Information

Below is the link to the electronic supplementary material.

Supplementary Material 1.

## Data Availability

Data will be available upon request from the corresponding authors.

## Abbreviations

DPZ: Donepezil HCl
PTX: Pentoxifylline
AD: Alzheimer’s disease
VaD: Vascular dementia
BBB: Blood brain barrier
ICH: International Conference on Harmonization
HPLC: High performance liquid chromatography
MoGAPI: Modified Green Analytical Procedure Index
AGREE: Analytical Greenness metric approach
GAC: Green Analytical Chemistry
WAC: White Analytical Chemistry
BAGI: Blue Applicability grade index
AchEIs: Acetylcholinesterase inhibitors
