# Supplementary material for: Whiteness and greenness metric tools for assessment of the spectrophotometric methods sustainability: mathematical manipulation of absorption spectra to resolve the interfering donepezil HCl and pentoxifylline in ethoniosomes
Source: BMC Chem. 2026 Mar 14;20(1):75. doi: 10.1186/s13065-026-01759-4 (PMC13063877; doi:10.1186/s13065-026-01759-4)
Supplement: Supplementary file 1 — Supplementary Material 1. [file 13065_2026_1759_MOESM1_ESM.docx]

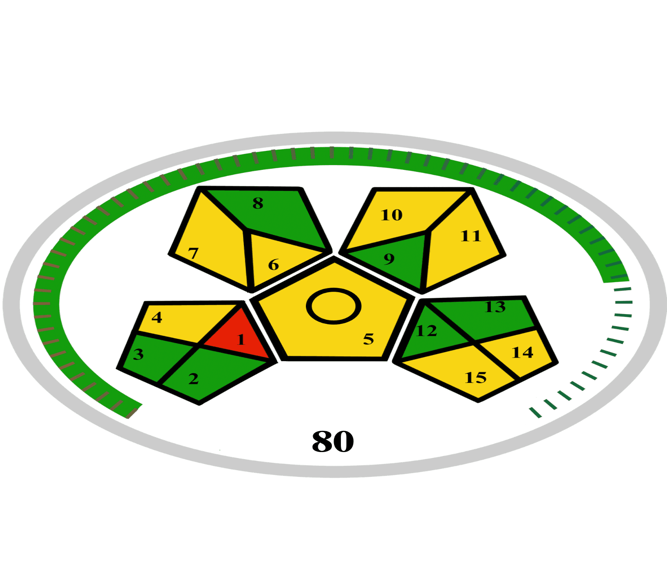


1. Sample treatment

2. Sample amount

3. Device positioning

4. Sample prep. stages

5. Automation, miniaturization


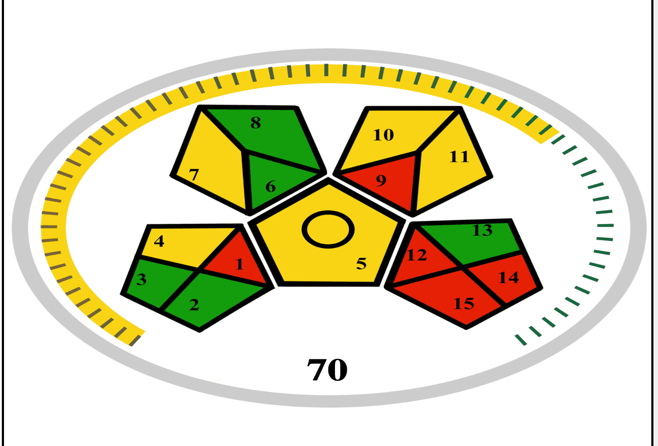
6. Derivatization

7. Waste

8. Analysis throughput

9. Energy consumption

10. Source of reagents

11. Toxicity

12. Operator's safety

Fig. S1 AGREE tool for greenness evaluation of the (a) proposed spectrophotometric method and (b) previously reported HPLC-MS method [30].


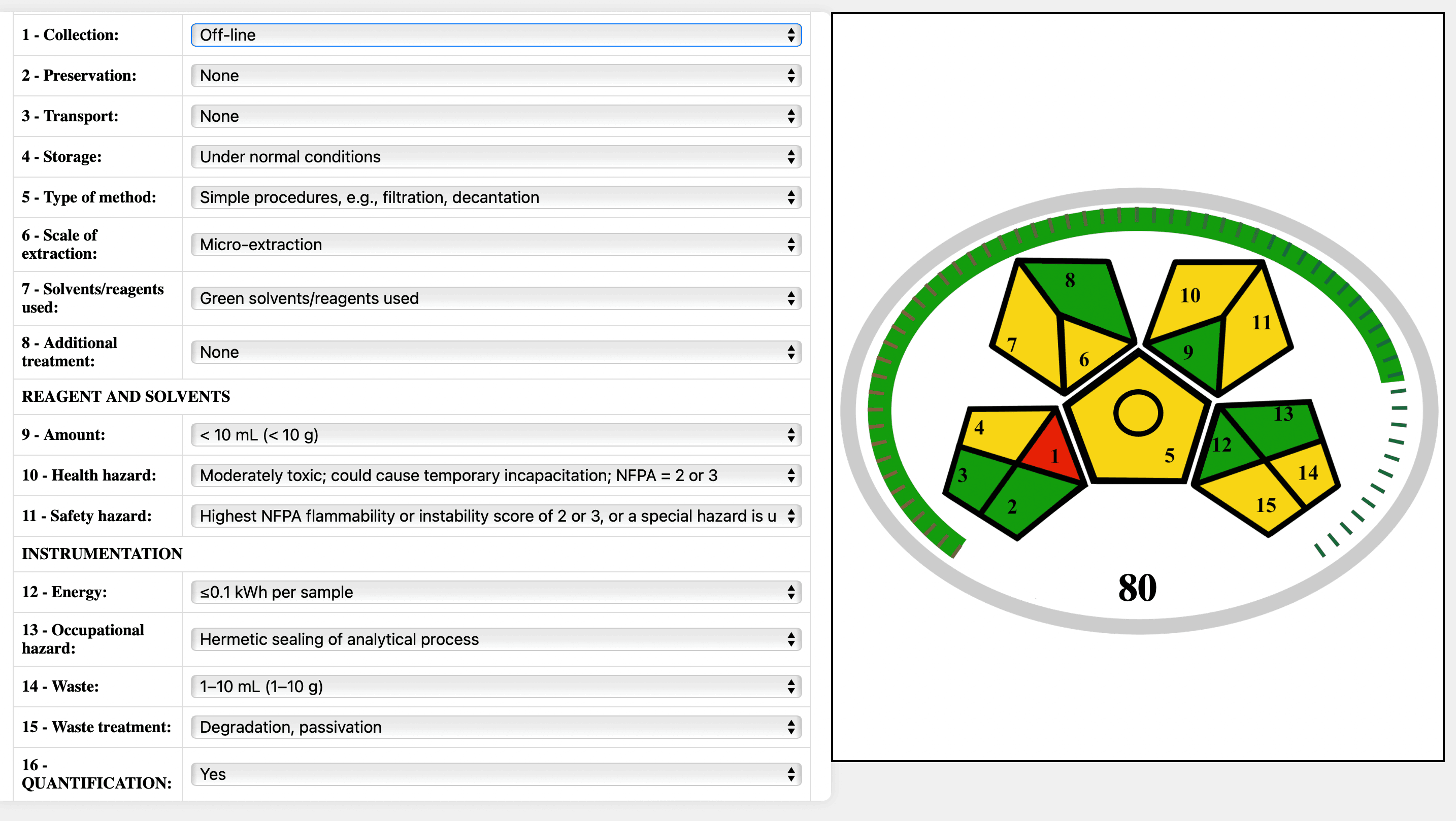


(a)


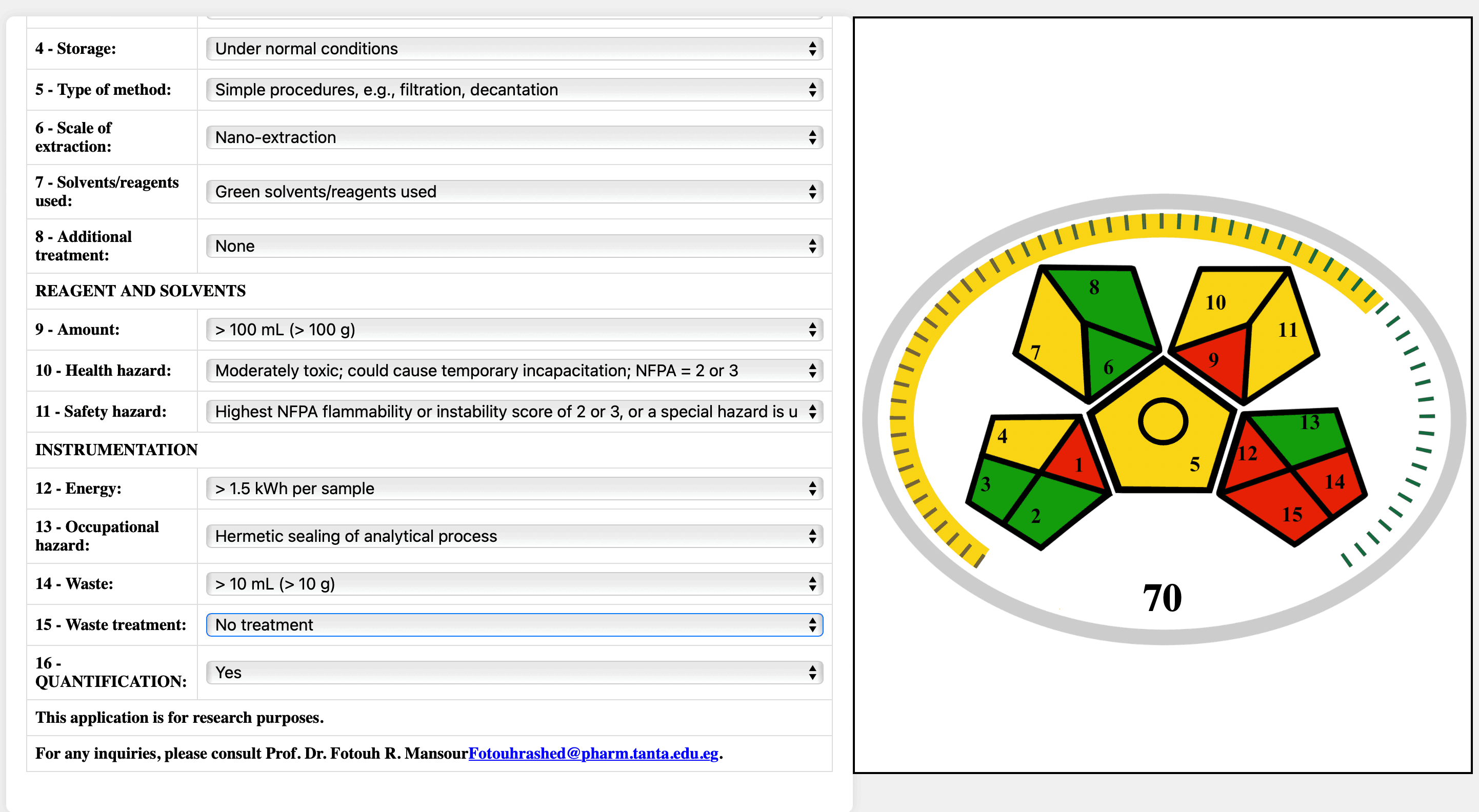


(b)

Fig. S2 MoGAPI tool for greenness evaluation of the (a) proposed spectrophotometric method and (b) previously reported HPLC-MS method [30].


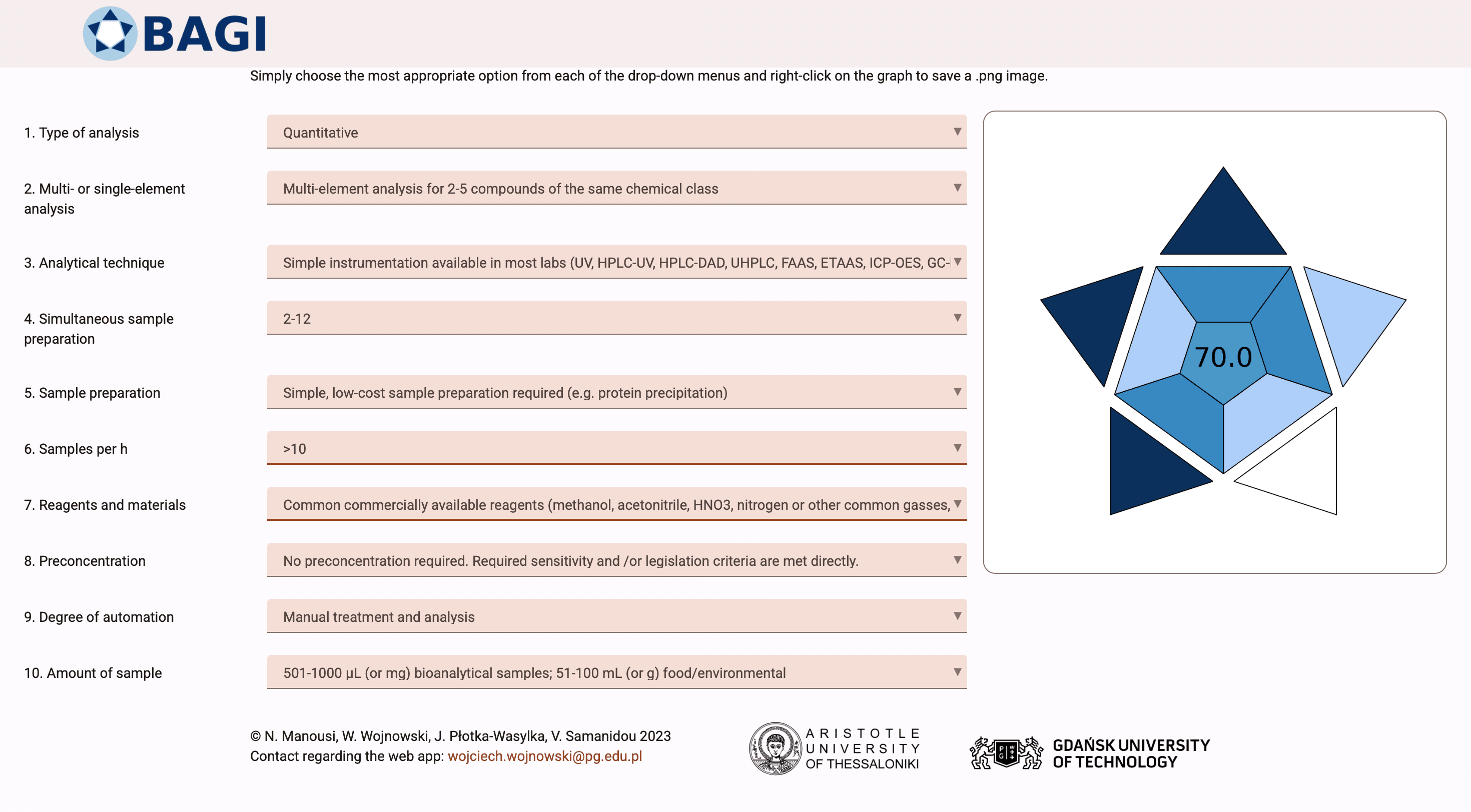

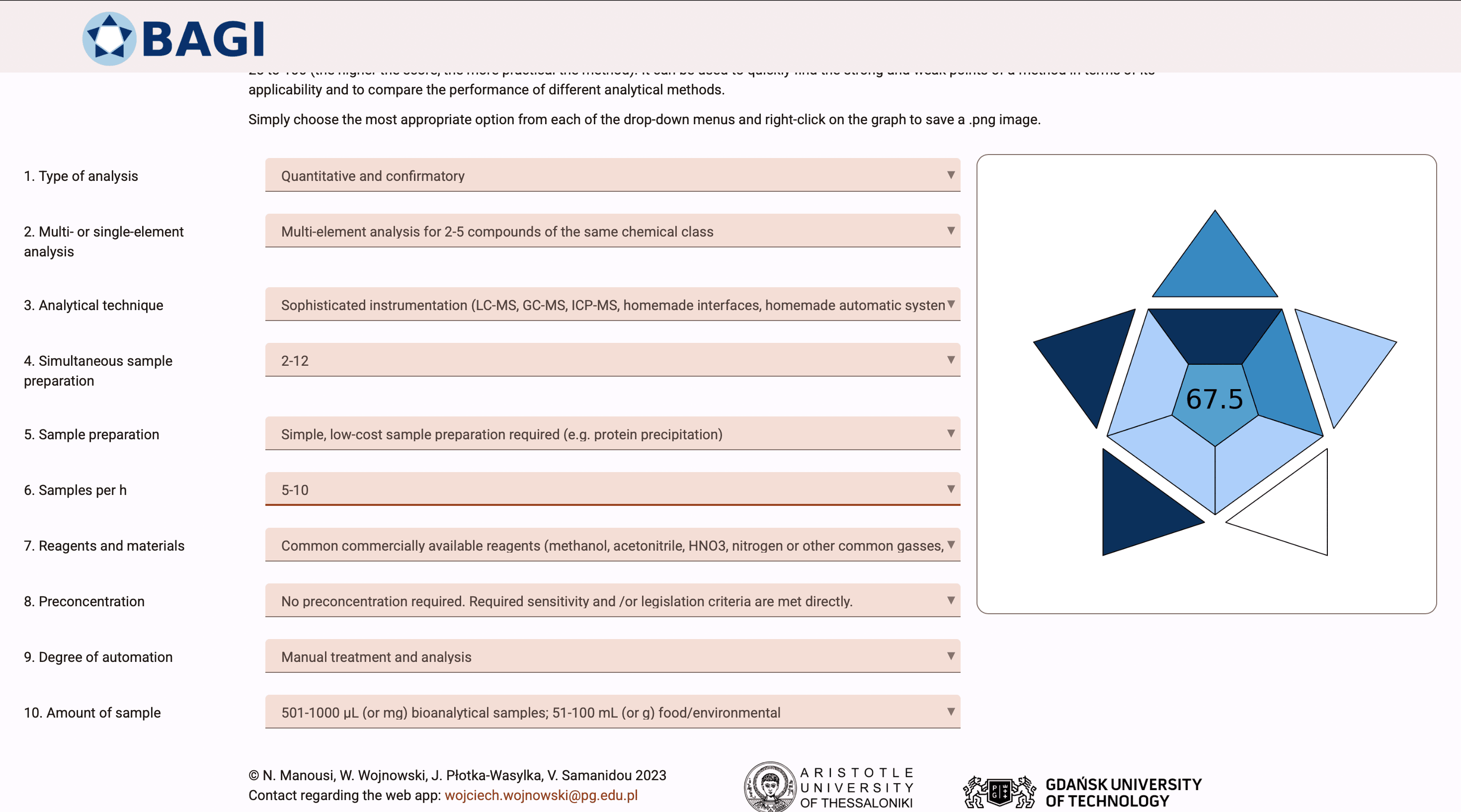


(b)

(a)

Fig. S3 BAGI tool for blueness evaluation of the (a) proposed spectrophotometric method and (b) previously reported HPLC-MS method [30].


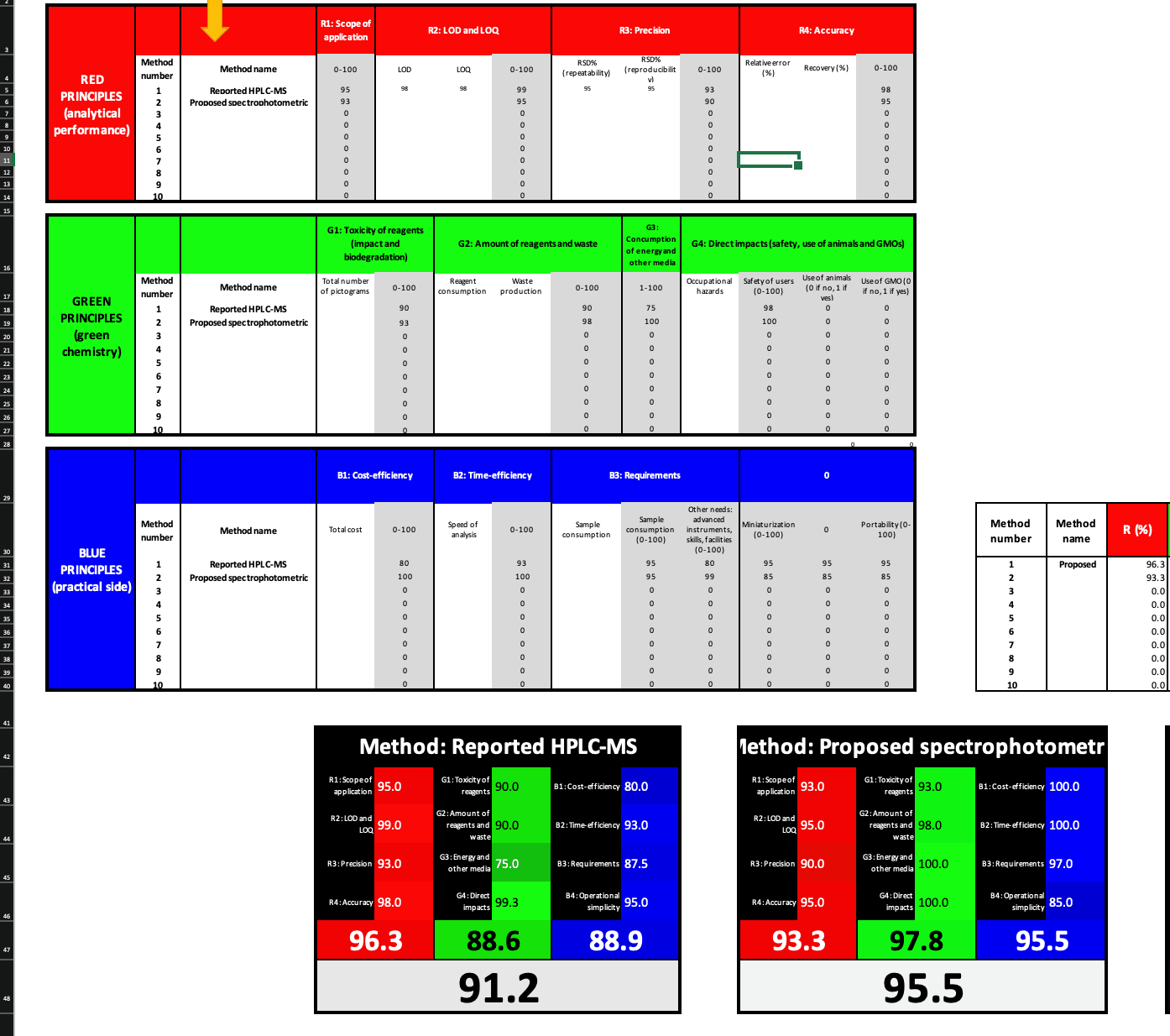


Fig. S4 excell sheet for whiteness evaluation of the proposed spectrophotometric method and previously reported HPLC-MS method [30].
